# Experimental Evidence of Generation and Reception by a Transluminal Axisymmetric Shear Wave Elastography Prototype

**DOI:** 10.3390/diagnostics11040645

**Published:** 2021-04-02

**Authors:** Antonio Gomez, Manuel Hurtado, Antonio Callejas, Jorge Torres, Nader Saffari, Guillermo Rus

**Affiliations:** 1 Department of Mechanical Engineering, University College London, London WC1E 6BT, UK; aj.gomez@ucl.ac.uk (A.G.); n.saffari@ucl.ac.uk (N.S.); 2 Department of Structural Mechanics, University of Granada, 18071 Granada, Spain; e.hurtadoestevez@go.ugr.es (M.H.); geresez@ugr.es (J.T.); grus@ugr.es (G.R.); 3 Instituto de Investigación Biosanitaria, ibs.GRANADA, 18012 Granada, Spain; 4 Excellence Research Unit “ModelingNature” (MNat), Universidad de Granada, 18071 Granada, Spain

**Keywords:** shear wave elastography imaging, transluminal elastography, phantoms, prostate

## Abstract

Experimental evidence on testing a non-ultrasonic-based probe for a new approach in transluminal elastography was presented. The proposed modality generated shear waves by inducing oscillatory rotation on the lumen wall. Detection of the propagated waves was achieved at a set of receivers in mechanical contact with the lumen wall. The excitation element of the probe was an electromagnetic rotational actuator whilst the sensing element was comprised by a uniform anglewise arrangement of four piezoelectric receivers. The prototype was tested in two soft-tissue-mimicking phantoms that contained lumenlike conduits and stiffer inclusions. The shear wave speed of the different components of the phantoms was characterized using shear wave elastography. These values were used to estimate the time-of-flight of the expected reflections. Ultrafast ultrasound imaging, based on Loupas’ algorithm, was used to estimate the displacement field in transversal planes to the lumenlike conduit and to compare against the readouts from the transluminal transmission–reception tests. Experimental observations between ultrafast imaging and the transluminal probe were in good agreement, and reflections due to the stiffer inclusions were detected by the transluminal probe. The obtained experimental evidence provided proof-of-concept for the transluminal elastography probe and encouraged further exploration of clinical applications.

## 1. Introduction

Elastography comprises a set of medical imaging modalities that evaluates tissue elasticity in order to obtain relevant pathological information [1,2]. In dynamic elastography, the dynamic nature of the excitation deforms the tissue, thus generating shear waves [3]. Shear waves are sensitive to changes in the tissue elasticity; therefore, their propagation is altered when a region of different stiffness is encountered [3,4]. In most cases, elastography has been performed from the outer surface of the human body, for instance, in breast cancer [5,6] and to evaluate chronic hepatic disease [7,8]. Transluminal elastography has not been extensively investigated, however, access via a lumen could provide feasibility advantages in situations where the target organ is deep in the body and performing elastography from an accessible body surface will remain challenging [3,9]. Most common examples of transluminal elastography could be found in cardiovascular imaging, for example, for the detection of vulnerable coronary atherosclerotic plaques [10] and the evaluation of the risk of rupture of carotid atherosclerotic plaques [11], as well as for prostate cancer detection using transrectal [12,13,14] and transurethral approaches [4,15].

Another transluminal approach, a transvaginal elastography device, developed by our group has been gaining relevance in the field of obstetrics and gynecology [16]. The transvaginal probe contained a cylindrical emitter that created torsional or axisymmetric waves that propagated through the cervical tissue. Piezoelectric sensors surrounding the disk emitter collected the signals, which then were postprocessed to calculate several mechanical properties related to the maturing process of the cervix during pregnancy [17,18,19]. Encouraged by the positive results achieved by the transvaginal project, we proposed in this article to extend the transvaginal design to a transluminal configuration. The new transluminal elastography probe used an electromechanically driven disk emitter to transmit an axisymmetric pattern of shear waves and an array of piezoelectric shear-mode receivers to sense the echoes generated after the propagation [4,20].

The main goals of this paper were as follows: (1) to provide experimental evidence on the capacity to generate axisymmetric shear waves in a transversal plane to the lumen by a transluminal emitter; (2) to provide experimental evidence on the capacity of piezoelectric-based transluminal receivers to sense the propagated shear waves. Feasibility of this transluminal approach, combined with the right inversion procedures, could open the door to several clinical applications where an accessible lumen is available and the mechanical properties of the surrounding tissue are of clinical relevance [21], for instance, in elasticity disorders in blood vessels [22] and to identify and grade prostate cancer [23,24]. This work showed the in-lab prototype transluminal probe tested in a soft tissue-mimicking medium for the first time, thus providing proof-of-concept for further research and development. First, the design and fabrication of the probe, the phantom fabrication, and the experimental setup were described. Then, results from ultrafast imaging of the transmitted shear waves and their interaction with a stiffer lesion-mimicking inclusion were shown. The readings from the receivers were analyzed and qualitatively compared with displacement fields estimated from ultrasound images. Finally, conclusions and proposed further lines of investigation were identified.

## 2. Materials and Methods

### 2.1. Transluminal Probe

**Figure 1:**
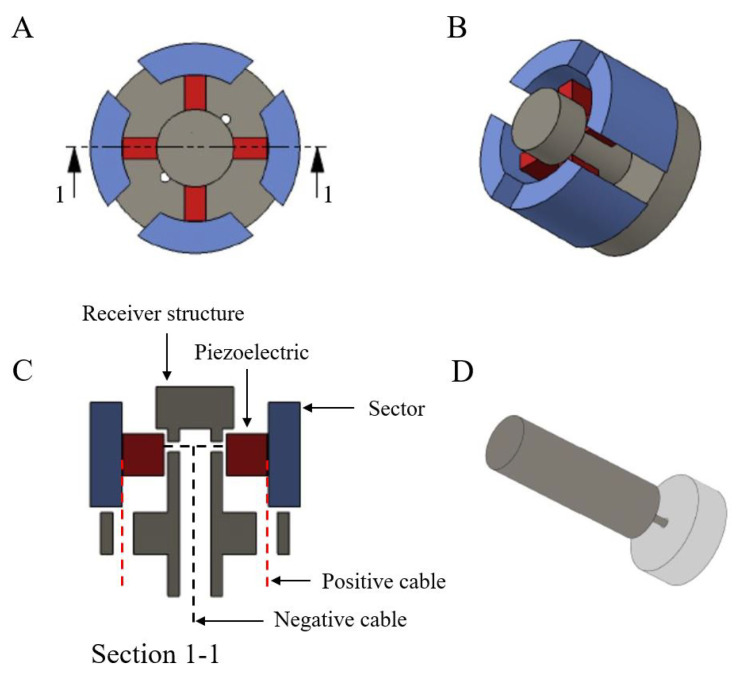
Schematic view of the two main components of the transluminal probe: (**A**) top view of the sensor showing the sectorized-anglewise ring (in blue), and the piezoelectric elements (in red); (**B**) isometric view of the assembled sensor; (**C**) sagittal cross-section of the sensor showing the wiring and supporting structure; (**D**) isometric view of the emitter disk (in light gray) and the electromechanical actuator (in dark gray).

A transluminal elastography probe, based on two main components, was designed and a prototype was created. The first component, the emitter part, consisted of a disk driven by an electromechanical actuator that transmitted a defined oscillatory rotation through its shaft. A Faraday cage enclosed the actuator to reduce the antenna effect, which is a source of spurious electrical cross talk. The second component, the sensing part, consisted of four transversely polarized piezoelectric ceramics (PZT-5), each one of them connected to a sectorized-anglewise ring. A centered rod served as a guide and support for the piezoelectric ceramics. Conductive silver resin was used to ensure electrical conductivity. The sensing part of the device was potentially able to gather spatial information and to discriminate the origin of the received signal, as the orientation of each receiver differed. Figure 1 shows a scheme of the prototype. All components were 3D-printed (Objet 30 Prime, Stratasys Inc., Eden Prairie, MN, USA) using a biocompatible photopolymer resin (MED610). For further details, the reader is referred to Patent P201730415 [20].

The maximum diameter of both emitter disk and sensor was 6 mm, a size that could suit many of the large blood vessels of the human body and other inner cavities such as the urethra and the cervix. Ensuring good mechanical contact between a transluminal fixed-diameter probe and a larger lumen is case-dependent. For instance, in the case of the urethra, suction can be applied, which would make the urethra wall collapse towards the probe [15].

The basic working principle of the proposed technique is explained here. The transluminal probe is introduced inside the lumen, with the emitter disk and the sectorized sensor contacting its inner wall. Then, the electromechanical actuator induces an oscillatory-rotation that is transformed into a source of shear wave radiation by the emitter disk. This form of transmission minimizes the emission of spurious compressional waves [25]. The wave propagates axisymmetrically along and from the lumen-wall into the bulk of the medium. The particle motion is transversal to the direction of wave propagation, tangential to the wave fronts, and contained in planes orthogonal to the luminal axis. Finally, the waves reaching the outer surface of the sectorized sensor transversely deform the piezoelectric ceramics, thus creating an electric potential difference that can be measured. The frequency of the resulting shear waves is linked to the frequency of the rotational oscillation of the emitter disk, which can be controlled. Nevertheless, the torque of the rotational actuator, the mechanical properties of the medium, and the mechanical contact condition between lumen and emitter disk may influenced the peak frequency and broadband of the resulting shear wave.

### 2.2. Tissue-Mimicking Phantom Fabrication

Soft-tissue-mimicking Polyvinyl Alcohol (PVA) phantoms containing stiffer inclusions were used for testing the transluminal probe. PVA concentration (PVA 99+% hydrolyzed, Sigma-Aldrich Chemical, St Louis, MO, USA) was chosen to achieve the range of shear wave speed usually found in soft tissues, which is in the order of a few m/s [3,26]. The targeted stiffness for the inclusions was in the order of two to three times that of the background material. For instance, these ranges were in agreement with observed values in prostate cancer [23,27,28]. The required percentage of PVA was added to distilled water at room temperature (21 ± 1 ${}^{\circ }$C). A mixing of graphite powder (Pressol Schmiergerate GmbH, Germany) and cellulose particles with a nominal diameter of 50 μm of (Type 50, Sigma-Aldrich Chemical, St. Louis, MO, USA) was added to increase acoustic scattering. Table 1 shows the proportion of ingredients. A sonicator (Fisherbrand 505, Thermo Fisher Scientific, Inc., Waltham, MA, USA) was used to homogenize the mixture. The energy amplitude was set at 60% of 500 W maximum, and the frequency at 20 kHz with cycles of 55 s on-time and 5 s off-time for 30 min. The final mixture was allowed to reach room temperature, and when visible bubbles disappeared it was poured into the chosen molds. The molds were 3D-printed using Polylactic Acid (PLA). After this, freezing–thawing cycles of 12 h at −17 ${}^{\circ }$C and 12 h at room temperature were applied. The final stiffness of the inclusion was tuned by increasing the number of cycles (see Table 1) [29].

Two different cylindrical phantoms were manufactured to compare the effect of different inclusion geometries on the reflection pattern. Both phantoms had a lumenlike conduit of 6 mm in diameter to host the transluminal probe components. The first phantom, phantom A (diameter of 60 mm and height of 60 mm), contained a prismatic inclusion of 10 × 20 × 60 mm, whose proximal face was 10 mm away from the lumen wall. The second phantom, phantom B (diameter of 40 mm and a height of 60 mm), contained a cylindrical inclusion of 5 mm in diameter and 60 mm length, whose proximal face was 8 mm away from the lumen wall.

### 2.3. Transluminal Transmission–Reception (T–R) Tests

**Figure 2:**
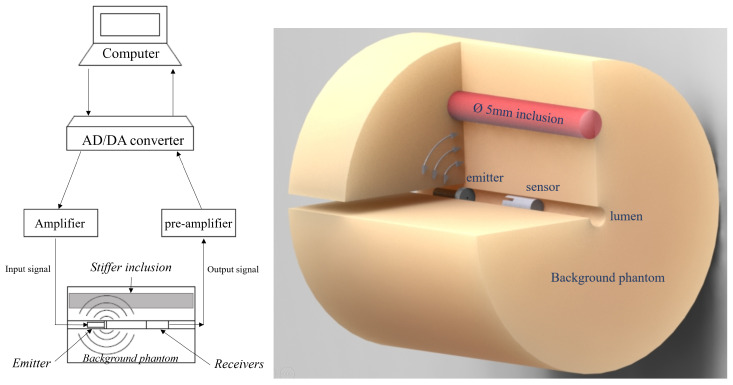
Experimental setup for the transluminal elastography tests. (**Left**): workflow for the transmission and reception, including a computer-controlled AD/DA converter with amplifier stages for both parts. (**Right**): 3D representation of the wave transmission for phantom B (inclusion in red color). Shear waves are pseudospherically transmitted from the emitter to the bulk of the phantom.

Transmission and detection of shear waves according to the proposed transluminal approach were studied using the the aforementioned phantoms. In this stage of study, and for the sake of simplicity, the emitter and sensor components of the transluminal probe were separated elements. In further stages, both components will be fused into a single catheterlike probe. The emitter and sensor were separately inserted into the phantoms through opposite ends of the lumenlike conduit. The distance between emitter and sensor in each phantom differed for visual convenience of the detected signals. In the case of phantom A, this separation was 10 mm; in the case of phantom B, it was 8 mm. The electronic system that generated the excitation and recorded the received signals consisted of a synchronized multichannel AD/DA converter with 24 bits and 192 kHz sampling rate. The experimental setup is shown in Figure 2. The excitation signal consisted of a single sinusoidal pulse of 800 Hz, which was amplified to 20 V peak-to-peak before reaching the emitter. A 5% duty cycle prevented fatal failure of the electromechanical actuator. The four receivers returned electrical signals directly to an array of preamplifiers before reaching the AD converter. A 5-kHz low-pass filter was applied to each received signal to eliminate high-frequency jitter. The same experiment was repeated three times and averaged to remove random noise. Time-of-flight estimations were performed by following the process detailed in [30]. First, a search window of several microseconds was selected. The time point where the signal first equals 80% of the local maximum amplitude was used to fit a sinusoidal signal with the frequency of the excitation, which allows the estimation of a theoretical start of the rising sine. This start time was taken as the time-of-flight of the received signal. All the elements were computer-controlled using high-speed communications ports and a Matlab environment (Release 2018b, The MathWorks Inc., Natick, MA, USA).

**Figure 3:**
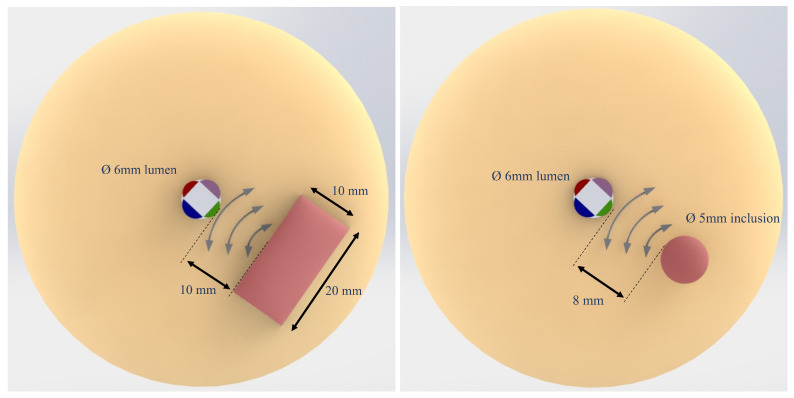
Schematic cross section of phantom A (**left**) and B (**right**) with the transluminal sensor inserted. Each receiver has been represented in a different color, where the green color indicates the element facing the stiffer inclusion, represented in red. Wave fronts of the reflected shear wave are shown as curved lines.

The position of the receivers, relative to the inclusion, was established as indicated in Figure 3). One receiver (green colored in the figure) was directly facing the inclusion, the next two receivers (magenta and blue colored) were arranged with a 90${}^{\circ }$ offset, and the remaining receiver was diametrically opposed to the inclusion.

### 2.4. Ultrafast Imaging for Tracking the Shear Wave Propagation and Characterization of the Shear Wave Speed

Ultrafast imaging by a Verasonics research system (Vantage 128, Verasonics Inc., Redmond, WA, USA) was used to track the shear-wave propagation generated by the emitter disk and, therefore, to validate the observations from the transluminal sensor. The final goal was to register the particle motion at a point close to the lumen wall at the plane where the sensor was located during the transluminal T–R tests.

**Figure 4:**
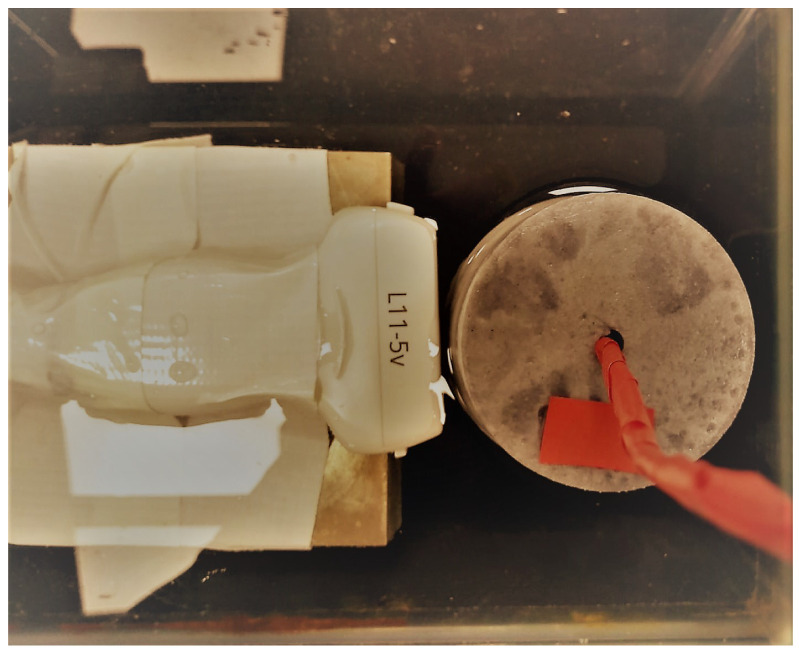
Experimental setup for ultrasound tracking of the shear waves generated by the transluminal emitter in phantom A. The ultrasound probe was located at the same plane where the transluminal sensor was placed during the transluminal Transmission–Reception (T–R) tests. The red rectangle shows the location of the stiffer inclusion.

The phantoms were partially submerged into a shallow water tank, thus allowing an accessible end of the lumen conduit to introduce the transluminal emitter. The ultrasound probe (linear array L11-5v, Verasonics Inc., Redmond, WA, USA) was entirely immersed in the water tank and kept fixed so its scanning plane was perpendicular to the lumen axis (see Figure 4). The emitter was placed at a distance of 10 mm for phantom A and 8 mm for phantom B, relative to the scanning plane of the ultrasound probe. These distances coincided with the separation between emitter and sensor during the transluminal T–R tests. The emitter disk and electromechanical actuator were not in contact with the water. The actuator was driven by the same setup used in the transluminal T–R tests (see Figure 2). The excitation was synchronized with the ultrafast imaging tracking through the external trigger of the Verasonics system. Ultrafast imaging was performed using plane-waves at a central frequency of 7.6 MHz and at 10 kHz frame acquisition rate. The whole process was repeated four times, allowing a waiting time between events of 200 ms, after which no reflections of previous events were recorded. Estimation of the particle motion was carried out by using the Loupas’ correlator algorithm to the output beamformed IQ data [31]. Finally, the displacement field as a function of time was obtained by averaging the previously estimated frames to remove the effect of random noise.

Characterization of the shear wave speed of the phantom was needed to estimate the arrival time for the different ways of propagation. For this, Shear Wave Elastography (SWE) [26] was performed in a similar configuration as described above. In this case, the excitation part was substituted by Acoustic Radiation Force (ARF) to remotely produce shear waves by means of ultrasonic excitation with a central frequency of 7.6 MHz and a duration of 1500 push cycles. The focal distance was set at a depth of 20 mm for the background parts and 10 mm for the inclusions. Immediately after the push sequence, the tracking events recorded the wave propagation, the particle motion was estimated, and the displacement field was averaged over four recording steps. A time-of-flight algorithm was implemented to estimate the group shear wave speed [32]. A rectangular ROI was selected at the push depth and averaged in depth, time data was upsampled by cubic interpolation and then the time of peak displacement was obtained. The slope of the linear fit between these times and the lateral distance yielded the group speed.

## 3. Results

### 3.1. Estimated Time-of-Flight

**Figure 5:**
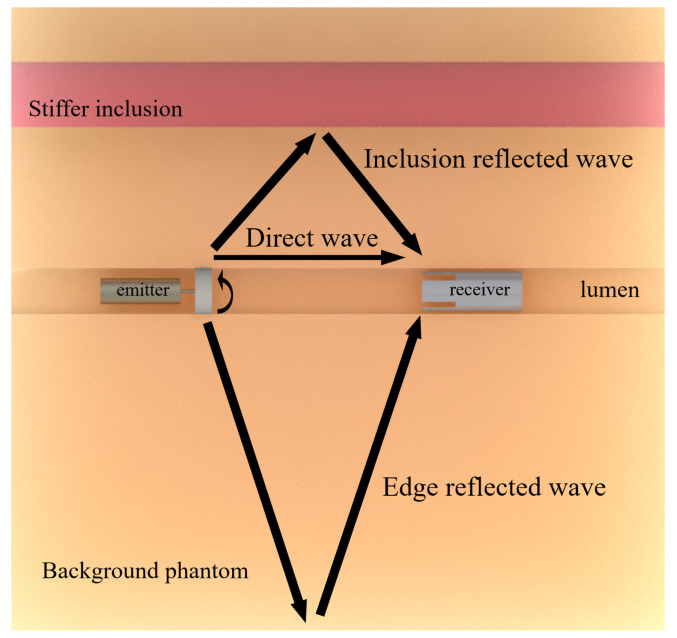
Scheme that depicts the three expected propagation paths for the transluminal T–R tests: direct wave, reflected wave against the inclusion, and the reflected wave against the outer surface of the phantom.

The shear wave generated by the transluminal emitter propagated axisymmetrically, thus generating pseudospherical wave fronts. The outer surface of the phantoms and the boundary of the inclusions generated reflections due to the change in shear acoustic impedance. This implied that different wave fronts reached the receivers at different times, depending on the length of the path followed. Figure 5 depicts the three different propagation paths expected: a direct propagation along the lumen wall, reflections from the boundary of the inclusion, and reflections from the outer surface of the phantom.

The shear wave speed of the background and inclusion materials were characterized using SWE to estimate the expected time-of-flight for the different wave fronts. Table 2 shows the group speed for the background and inclusion materials. The time-of-flight in each phantom was estimated given the length of the propagation paths and the corresponding speeds (see Table 3).

### 3.2. Transluminal T–R Tests

**Figure 6:**
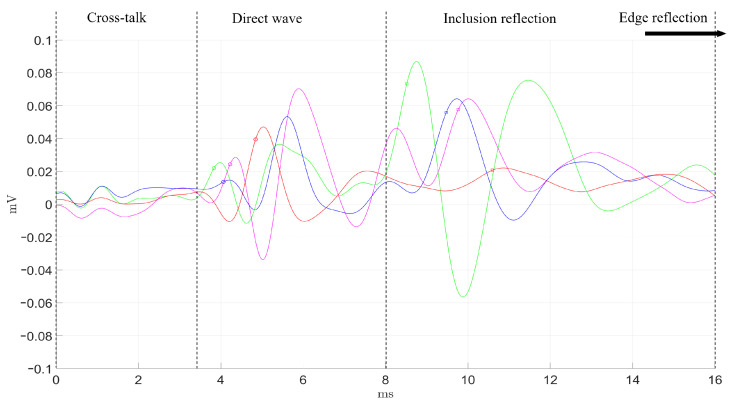
Readout from the four sensors (color code in Figure 3) and expected time-of-flight of the different paths of wave propagation in Phantom A. The vertical dashed lines represent the estimated time-of-flight calculated from the shear-wave velocity. The reflected wave against the outer surface of the phantom was not recorded due to a technical compromise between time resolution and the limited memory acquisition of the equipment used.

**Figure 7:**
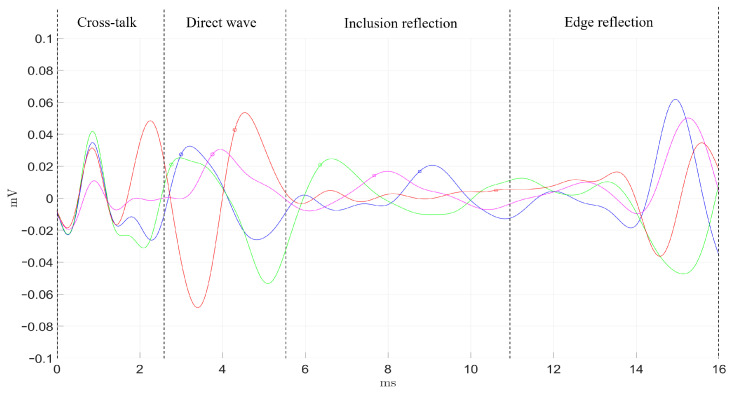
Readouts from the four receivers (color code in Figure 3) and expected time-of-flight of the different paths of wave propagation in Phantom B. The vertical dashed lines represent the estimated time-of-flight calculated from the shear-wave velocity.

As described in the previous section (Section 2.3), the transluminal emitter and sensor were inserted through the lumenlike conduit from opposite apertures. The detected signals by the four receivers are shown in Figure 6 and Figure 7, following the color code established in Figure 3. Time-of-flight estimations for each propagation path are shown as vertical dashed lines. The maximum signal amplitude, in terms of peak-to-peak voltage, was 20 V for the transmission and 40–100 μV for the reception.

In the readouts from both phantoms, the four receivers showed a series of initial perturbations from 0 ms to 2.5 ms, that were due to spurious electrical cross talk. This phenomenon was more noticeable in phantom B. The direct wave propagation was clearly observable between the times of 3.1 and 8.0 ms for phantom A. In phantom B, the direct wave was detected in an earlier time range, from 2.5 to 5.6 ms, as the separation between emitter and sensor was 2 mm shorter. The dissimilar orientation of each receiver related to the position of the inclusion yielded several observations at the time region where the reflection coming from the inclusion was expected. First, the receiver facing the inclusion (green) detected the first peak at the earliest time, which is reasonable as the receiver–inclusion distance was the shortest. Second, blue and magenta receivers detected the reflection slightly later, which can be related to the increment in distance. Amplitudes of both signals were similar, and slightly lower compared to that of the green receiver. The time delay observed for the direct wave between both blue and magenta signals could be partially due to minor misalignments in the orientation angle of the sensor respecting the inclusion. With a propagation speed of 3.2 m/s, every additional millimeter in the propagation path would imply an additional time delay of 0.312 ms. Third, the detected signal by the red receiver showed no clear peak waves associated to the reflection, which seemed correct as the receiver was located on the shaded region with respect to the inclusion reflections. Finally, the four receivers detected the reflection coming from the outer surface of the phantom starting at 10.9 ms in phantom B. This reflection occurred at 17 ms for phantom A, which is larger in diameter than phantom B, however, it could not be recorded. Due to the compromise between time resolution and the limited memory acquisition of the equipment used, the maximum time recorded was 16 ms.

The four receivers were tested at four different positions to assess the performance consistency. Green, blue, and magenta receivers seemed to perform similarly, while the red one did not in all of the tested cases. This was particularly noticeable in the direct wave time region (see Figure 6 and Figure 7). This dissimilarity can be attributed to the inherent variability of the handmade assembling of the probe, where differences in the positioning of the piezoelectric ceramics and the distribution of the conductive silver resin have a strong impact on the final performance of each receiver. Further improvements on the assembling process must be pursued in future work.

Overall, good agreement between expected time-of-flight and readouts from the receivers was achieved. Minor delays in the signals and amplitude differences can also be attributed to the variability associated to the assembling process. In addition, when comparing results from both phantoms, it can be seen that the larger inclusion from phantom A produced peaks for the reflected wave of almost twice the amplitude of those produced in phantom B. Clearly, the difference in size and in the geometry of the interface between background and inclusion had an impact. The larger and planar surface of the inclusion in phantom A reflected more energy and reduced the attenuation due to geometric dispersion of the reflected wave.

### 3.3. Transluminal Wave Propagation Tracking

**Figure 8:**
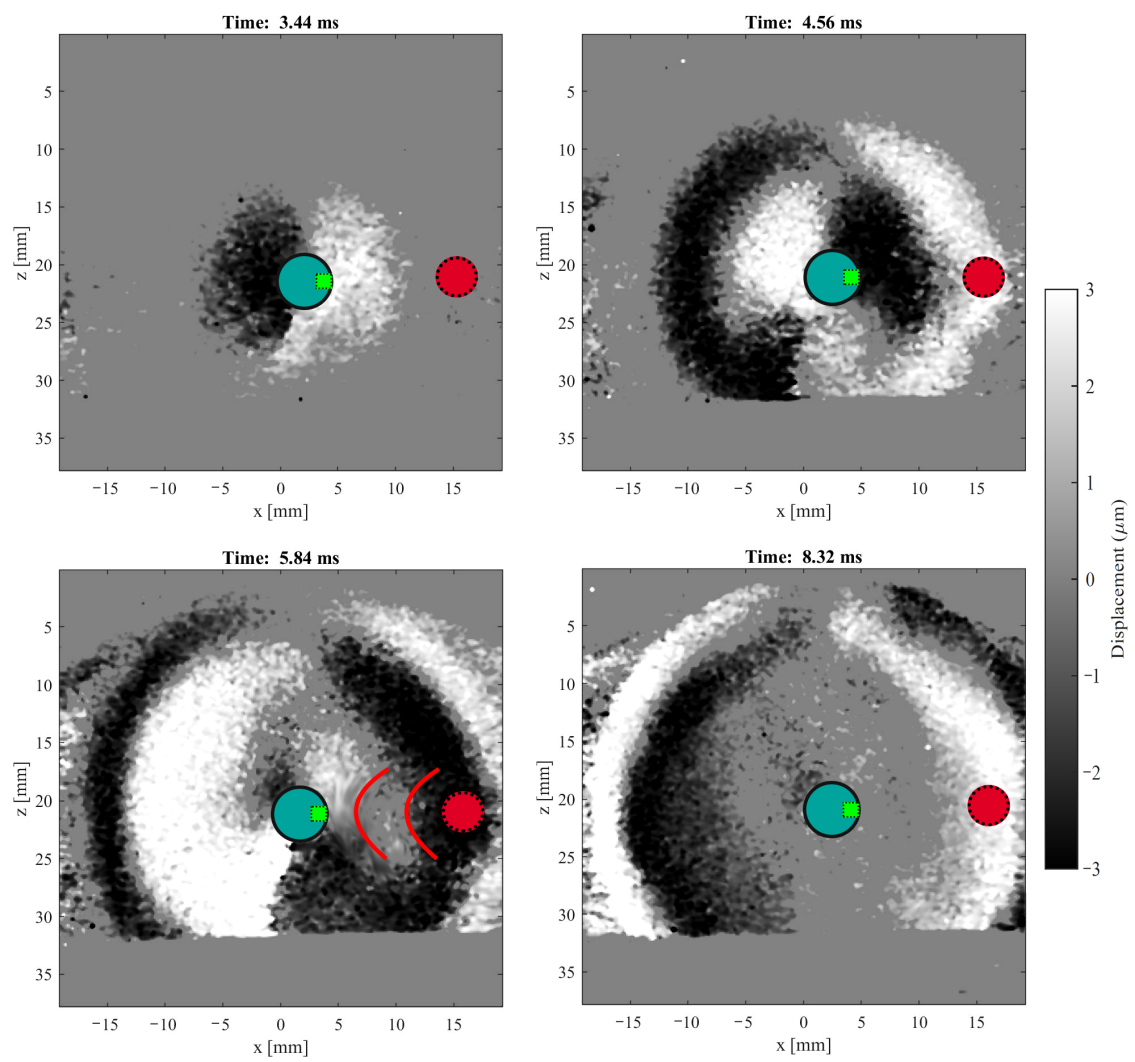
Example of ultrasound images at different times on the cross-section plane that contains the transluminal sensor (turquoise circle) in phantom B. The green sensor is facing the inclusion (red circle). The ultrasound probe scanned from the upper side of the figures.

Figure 8 illustrates an example of ultrasound images at different propagation times for tracking of the shear wave generated by the transluminal emitter in phantom B. The images correspond to a cross-section plane of the phantom that contains the transluminal sensor. The ultrasound probe performed imaging from the upper side of the figures. The propagation was clearly visible, as well as the reflection generated against the inclusion (visible in the snapshot taken at time 5.84 ms). The magnitude plotted in Figure 8 is the projection of the particle motion over the direction of the ultrasound beam. The value of this projection is maximal (in the order of a few micrometers), where the particle motion is aligned to the ultrasonic beam, that is, the radial line parallel to the face of the ultrasound transducer that goes through the center of rotation for the emitter disk. On the contrary, it is nearly zero where the particle motion is totally perpendicular to the ultrasound beam. This behavior demonstrates that the particle motion was tangential to the wave fronts and transversal to the direction of propagation, which is the case for shear waves.

**Figure 9:**
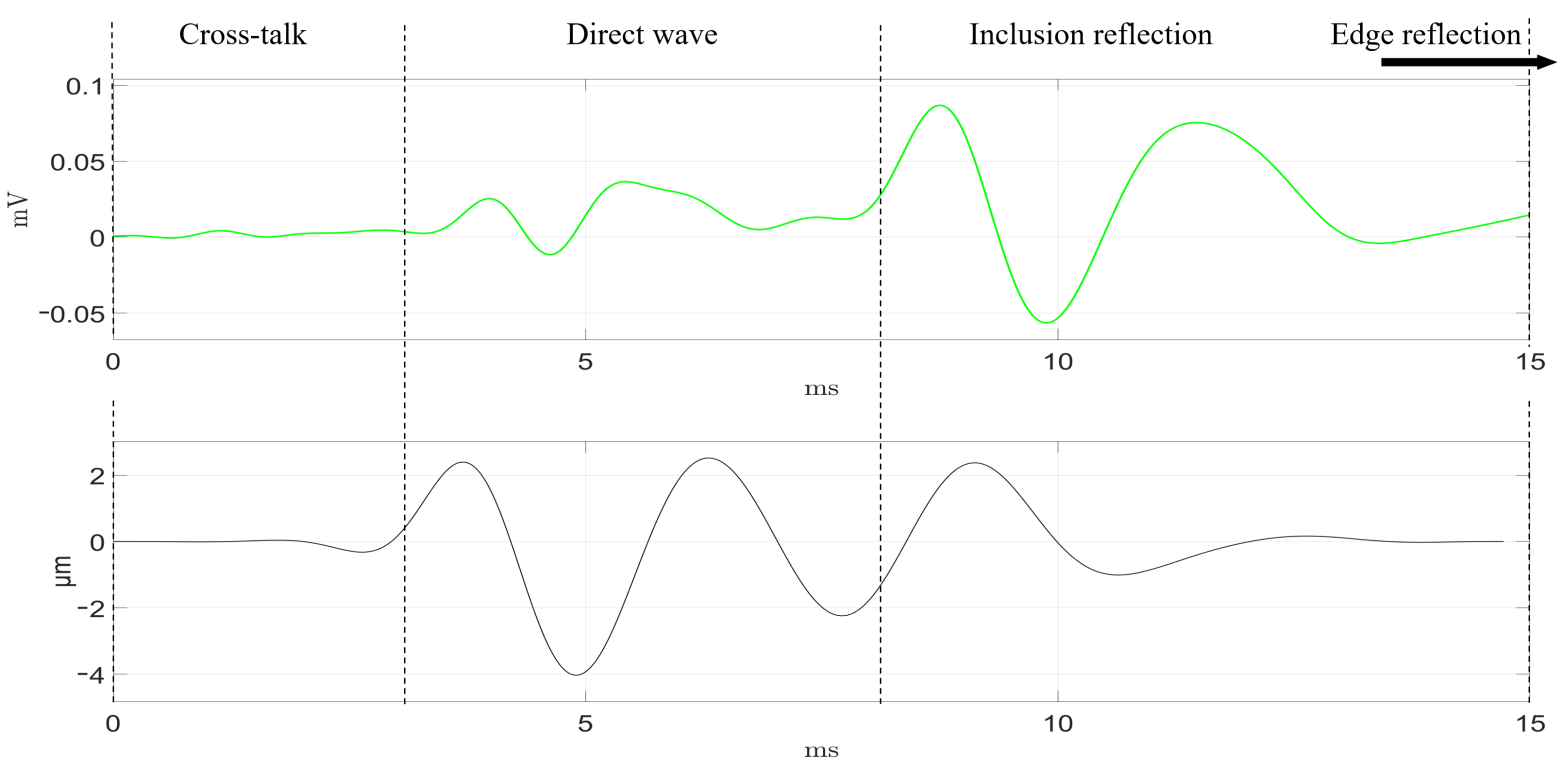
Readout (in green) from the green receiver and the reconstructed displacement (in black) from the ultrasound imaging at a proximal region to the sensor, in phantom A.

**Figure 10:**
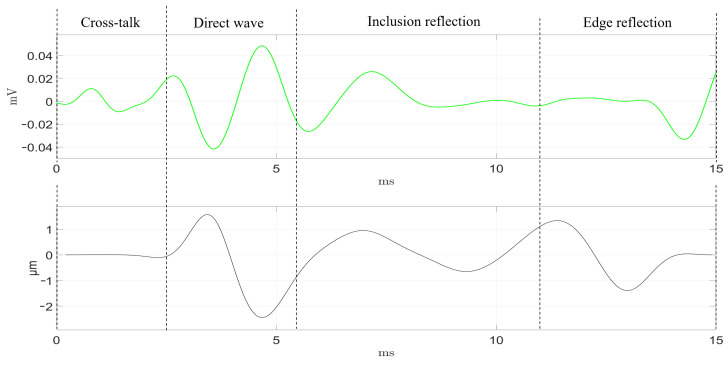
Readout (in green) from the green receiver and the reconstructed displacement (in black) from the ultrasound imaging at a proximal region to the sensor, in phantom B.

Figure 9 and Figure 10 compare the readout from the green sensor (in green color) against the reconstructed displacement from the ultrafast imaging measurements (in black color) at a proximal region to the sensor in both phantoms. In both phantoms, and neglecting the initial cross-talk from the receiver’s readout, the start time of the direct wave was similar to those detected by the green receiver. The shape of both signals along the whole duration of the test was particularly similar in phantom A. In phantom B, the mismatch was slightly higher. However, in both phantoms, the first peak from the inclusion reflection was clearly observable and coincided with the ultrasound measurements. This supports the research goal that detecting reflections from a stiffer inclusion is feasible.

## 4. Discussion

In this paper, a new transluminal elastography probe prototyped by our group has been presented. The probe had separated emitter and sensor elements. The emitter was based on an electromechanically driven disk emitter and the sensor was composed of an array of four piezoelectric shear-mode receivers. The emitter generates a pseudospherical pattern of shear waves that are reflected after encountering areas of altered mechanical properties. These reflections can be analyzed to obtain information regarding certain clinical features of the areas of altered mechanical properties, for instance, the location and the change in viscoelastic properties.

The transluminal set was tested on soft-tissue-mimicking media containing stiffer inclusions. Shear wave speed of both the background material and inclusion of the phantoms were characterized using SWE implemented in a Verasonics Vantage system. Expected time-of-flight for the different propagation paths direct, reflection against the inclusion, and reflection against the outer surface of the phantom was calculated.

Qualitative comparison between the expected time-of-flight and the signals captured by the array of sensors was carried out (Figure 6 and Figure 7). Amplitude peaks related to the different propagation paths were observed. Overall, the time signature of the received signals was in good agreement with the time-of-flight estimations. Time lags among the signals registered by the four receivers were attributed to the difference in relative position with respect to the inclusion and the variability and limited consistency in the by-hand assembling of each receiver. This variability was verified after observing that the performance of each receiver was consistent at different angular positions within the lumen. This was particularly noticeable for the red receiver, whose performance differed the most compared to the rest. Furthermore, the spurious electrical cross-talk was more noticeable in phantom B in all the tests. The phantoms needed to be dried prior to the tests as they were stored in water for a few hours. Different levels of humidity in the lumen before testing was believed to cause the discrepancy in the amplitude of the cross-talk.

The resulting peak frequency of the transmitted shear waves was around 400 Hz despite the electromechanical actuator being driven by a single sine of 800 Hz. As long as greater attenuation is assumable, higher frequency and a shorter pulse duration would increase the time lag between the different propagation paths, thus making the distinction between them clearer. However, achieving this was not a simple task. The conditions of the mechanical contact between the disk and the lumen wall, the viscoelastic properties of the medium, and the torque provided by the actuator play a critical role in the resulting frequency and shape of the transmitted oscillation. Research on the contact mechanics and improvements in the transmission technology must be addressed in future investigations.

It is important to remark that in a real situation, where the properties of the medium of propagation are unknown, inverse procedures will be required to estimate the time-of-flight and the location of the inclusions. This article aims to provide an experimental proof-of-principle, which lays the foundation for further developments.

Other limitations were identified. By using group velocity, it is only possible to obtain elasticity parameters under homogeneous and linear simplifications. A frequency sweep would provide a more complete map of the frequency-dependence of the velocity and attenuation, yielding to dispersion curves from which complex viscoelastic properties with potential clinical use could be derived [2].

Ultrasound imaging by the same Verasonics Vantage system was used to compare against the readout from the green receiver facing the inclusions. For the ultrasound images, the displacement field generated by the transluminal emitter was reconstructed using a Loupas’ correlator algorithm. The direct propagation and reflection against the inclusions and the outer surface of the phantoms were evidenced (see Figure 8). The variation of the amplitude registered by the ultrasound images along the scanning plane, with maximum values where the particle motion was fully aligned with the direction of the ultrasound beam, demonstrated that the type of wave generated by the transluminal emitter was shear. Particle motion from a spatial point near the location of the green receiver was registered. Comparison between the readout from the green receiver and the ultrasound measurements demonstrated a relative degree of agreement, particularly for the start times of the direct wave and the reflection against the inclusion. Nevertheless, this must be analyzed carefully, as some differences between the two techniques of measurement can provide bias in the comparison. For instance, the spatial resolution of the ultrasound reconstruction was much finer than that of the transluminal sensor, as the receivers sensed the incident wave over the whole surface of the sectorized ring. A large receiver has limited ability to discriminate signals in space. In future work, further improvements need to be accomplished to improve the spatial performance of the sensor, as well as to establish a fairer and more robust validation approach. For example, the spatial resolution would be improved by increasing the number of receivers and optimizing its size.

It is worth noting the difference in sampling frequency between ultrafast ultrasound imaging, 10 kHz, and the transluminal sensor, 192 kHz. In principle, this higher sampling frequency would allow detecting shear waves in media with higher wave speed.

The amplitude of particle displacement generated was in the order of a few micrometers (Figure 9 and Figure 10), which is in agreement with values from other elastography applications that are in clinical use and, therefore, are safe [3]. With these values, the range of strain was within the linear elastic regime of most soft tissues, this is below 5–10%, which is under the threshold for irreversible damage [33]. In future stages of development, the transluminal probe will be assembled forming a catheter. Direct physical contact, and therefore, patient discomfort will have to be addressed specifically for each clinical application.

The experimental observations in soft-tissue phantoms provided proof-of-concept on the feasibility of transluminally generating shear waves and on the ability of detecting echoes from regions of altered elasticity. This outcome encourages further investigation and analysis of potential applications, for instance, the evaluation of the risk of lipid cap rupture in atherosclerotic plaques in large vessels and the transurethral identification of stiff cancerous nodules in the prostate.

## Figures and Tables

**Table 1:** Weight percentage (*w*/*w*) of the ingredients for fabricating the background phantom and inclusion.

	Background	Inclusion
PVA	15%	20%
Graphite powder	0.5%	0.5%
Cellulose	0.5%	-
Freezing–thawing cycles	1	2

**Table 2:** Values for the shear wave speed (mean ± sd) for the background and the inclusion in both phantoms.

Phantoms A and B	Shear Wave Speed (m/s)
Background	3.20 ± 0.18
Inclusion	7.31 ± 0.16

**Table 3:** Time-of-flight estimation for the direct wave, the reflection against the inclusion, and the reflection against the outer surface in both phantoms.

	Phantom A		Phantom B	
	**Distance (mm)**	**Time (ms)**	**Distance (mm)**	**Time (ms)**
Direct wave	10	3.1	8	2.5
Inclusion reflected wave	25	8	18	5.6
Edge reflected wave	55	17	35	10.9

## Abbreviations

The following abbreviations are used in this manuscript:

PVA	Polyvinyl Alcohol
PLA	Polylactic Acid
PRF	Pulse Repetition Frequency
SWE	Shear Wave Elastography
ARF	Acoustic Radiation Force
T–R	Transmission–Reception
FFT	Fast Fourier Transform
	
